# NUMBER OF CASES OF VARICELLA AND HOSPITALIZATION IN A PEDIATRIC REFERENCE HOSPITAL IN BRAZIL AFTER INTRODUCING THE VACCINE

**DOI:** 10.1590/1984-0462/2021/39/2019215

**Published:** 2020-07-03

**Authors:** Henrique Botelho de Abreu e Silva, Isabella Cristina Tristão Pinto, José Geraldo Leite Ribeiro, Leonardo Santos Resende, Ludymila Keren de Carvalho, Marcelle Marie Martins Maia, Roberto Ferreira de Almeida Araújo, Lilian Martins Oliveira Diniz

**Affiliations:** a Faculdade Ciências Médicas de Minas Gerais, Belo Horizonte, MG, Brazil.; b School of Medicine, Universidade Federal de Minas Gerais, Belo Horizonte, MG, Brazil.; c Hospital Infantil João Paulo II, Belo Horizonte, MG, Brazil.

**Keywords:** Chickenpox, Chickenpox vaccine, Varicella zoster virus infection, Varicela, Vacina contra varicela, Infecção pelo vírus da varicela-zóster

## Abstract

**Objective:**

To assess the number of cases and the profile of hospitalizations from varicella after the introduction of the measles, mumps, rubella and varicella combination vaccine in the public health system.

**Methods:**

Retrospective study in an infectious diseases pediatric hospital of reference in Southeast Brazil. The cases with a clinical diagnosis of varicella, from January 2011 to June 2016, were assessed from pediatricians’ medical records. The hospitalizations were classified into a pre-vaccine group and post-vaccine group, based on the date the vaccine was introduced (September 2013). Both groups were compared by age, sex, time of hospitalization, reason for hospitalization, hospital complications, duration of intensive care, and clinical outcome.

**Results:**

A total of 830 hospitalizations were recorded; 543 in the pre-vaccine period and 287 in the post-vaccine period, a reduction of 47.1% (p<0.001). In both periods, a similar profile in the hospitalizations was noticed: majority male; aged between one to five years old; most complications due to secondary causes (mainly skin infections); main outcome was clinical improvement and discharge from the hospital. In the pre-vaccine period, six deaths were recorded and two were recorded in the post-vaccine period.

**Conclusions:**

The profile of the hospitalizations was expected to stay the same since this study did not compare vaccinated with unvaccinated children, but hospitalizations before and after the vaccine was introduced. In accordance with the medical literature, we found a significant fall in the number of hospitalizations from varicella.

## RESUMO

**Objetivo:**

Avaliar o número de casos e o perfil das internações por varicela após a introdução da vacina quádrupla viral na rede pública.

**Métodos:**

Estudo retrospectivo conduzido em hospital pediátrico referência em doenças infectocontagiosas na Região Sudeste do Brasil. Foram avaliados os casos com diagnóstico clínico de varicela, registrados em prontuário por médico pediatra, de janeiro de 2011 até junho de 2016. As internações foram classificadas em grupo pré-vacinal e grupo pós-vacinal, com base na data de introdução da vacina (setembro de 2013). Os grupos foram comparados em relação a: faixa etária, sexo, tempo de hospitalização, causas da internação, complicações hospitalares, tempo da internação em terapia intensiva e desfecho clínico.

**Resultados:**

Foram documentadas 830 internações, 543 no período pré-vacinal e 287 no pós-vacinal, ocorrendo redução de 47,1% nas internações (p<0,001). Em ambos os períodos, notou-se um perfil similar das internações, predominantemente: sexo masculino; faixa etária de um a cinco anos; por causas secundárias (principalmente infecções de pele); evoluindo com melhora clínica e alta hospitalar. Em relação ao número de óbitos, ocorreram seis no período pré-vacinal e dois no pós-vacinal.

**Conclusões:**

A manutenção do perfil das internações era esperada, visto que o trabalho não comparou crianças vacinadas com não vacinadas, e sim internações pré e pós-vacinais. Observou-se, em concordância com a literatura, queda substancial no número de internações por varicela.

## INTRODUCTION

Chickenpox is a very contagious infectious disease that is relevant around the world. ^1^ ^,^ ^2^ ^,^ ^3^ ^,^ ^4^ Annually, it generates around 4.2 million hospitalizations for serious complications worldwide.^4^ In Brazil, between 2012 and 2017, 602,136 cases were reported and 38,612 hospitalizations related to the disease were registered, affecting mainly the age group between one and four years old.^5^

Due to primary varicella-zoster virus (VZV) infection, chickenpox is manifested by cutaneous and mucosal lesions and is associated with nonspecific systemic signs and symptoms.^1^ ^,^ ^2^ ^,^ ^3^ The lesions are pruritic, with a centripetal distribution and regional polymorphism. ^1^ ^,^ ^3^ Initially, blotches appear that then become papules, vesicles and, later on, scabs. ^1^ ^,^ ^2^ ^,^ ^3^ The diagnosis is clinical.^3^ Confirmation occurs through viral isolation in a culture or through a polymerase chain reaction.^2^

Although it is generally benign, chickenpox can develop with major morbidity and mortality complications. These cases are more common in children under one year of age, who are malnourished and immunocompromised.^3^ Secondary infection of the skin from pyogenic bacteria (*Streptococcus pyogenes*, *Staphylococcus aureus*) is the most common complication.^6^ This type of infection makes the patient at risk for sepsis and focal infections, such as pneumonia.^3^ The risk of developing visceral complications is higher in immunocompromised patients (30-50%), and there is a 15% lethality rate when no treatment is performed.^2^ In children, complications associated with the central nervous system (CNS) are the second leading cause of hospitalization for chickenpox.^3^ ^,^ ^6^ Other complications include: myocarditis, nephritis, arthritis, Reye’s syndrome, hepatitis and ophthalmic lesions.^2^

Conservative estimates show that chickenpox is responsible annually for 4,200 deaths worldwide.^4^ ^,^ ^7^ Mortality rates are lower than those of other vaccine-controlled diseases, both in the pre-vaccination and post-vaccination era.^8^ Despite this fact, the disease has a significant impact on the population, as it has a severe outcome in several population groups. As such, it is important to act in order to prevent it.^4^

Nowadays, the use of vaccines is adopted in many countries as an important strategy for the prophylaxis of chickenpox.^9^ ^,^ ^10^ ^,^ ^11^ ^,^ ^12^ ^,^ ^13^ ^,^ ^14^ ^,^ ^15^ ^,^ ^16^ ^,^ ^17^ The chickenpox vaccine was developed by Takahashi, in 1974, and is composed of attenuated viruses, made from the Oka strain.^3^ ^,^ ^9^ Seroconversion with a vaccine dose ranges from 85 to 100% in previously healthy children between 12 months and 12 years old.^6^ The Ministry of Health, through the National Immunization Program (*Programa Nacional de Imunizações* - PNI), implemented the chickenpox vaccine in the National Vaccination Calendar in September 2013. This vaccine is given in conjunction with the measles, rubella and mumps vaccines, in the tetra viral vaccine. The child receives the triple viral vaccine (measles, rubella, mumps) at 12 months and the tetra viral vaccine at 15 months of age. As of 2018, the second dose of the chickenpox vaccine began to be administered at the age of four.^1^ ^,^ ^18^ However, during the period evaluated in this study, the vaccine was only available for the age group of 15 up to 24 months, in a single dose.^19^

The vaccination against chickenpox dramatically changed the epidemiology of the infection, reducing its incidence in sentinel communities.^2^ National and international publications report that the use of the vaccine has had a favorable impact, including: the reduction of hospitalizations, serious outcomes and hospital expenses, and the protection of non-immunized herds.^9^ ^,^ ^10^ ^,^ ^11^ ^,^ ^12^ ^,^ ^13^ ^,^ ^14^ ^,^ ^15^ ^,^ ^16^ ^,^ ^17^ In a study carried out in Brazil, there was a 37.9% reduction in hospital expenses three years after the introduction of the vaccine and it is expected that this savings will reach 80% in 30 years.^17^ ^,^ ^20^

In Brazil, only severe hospitalized cases and death from chickenpox are compulsory to report.^1^ Therefore, studies to assess the variation in the incidence of the disease after the introduction of the vaccine should be based mainly on hospital data. Considering this context, the present work proposed to evaluate the number of cases and the profile of hospitalizations for chickenpox at the João Paulo II Children’s Hospital (*Hospital Infantil João Paulo II* - HIJPII), which is part of the Hospital Foundation of the State of Minas Gerais (*Fundação Hospitalar do Estado de Minas Gerais* - FHEMIG), after the introduction of the tetra viral vaccine in the PNI. The HIJPII is located in the city of Belo Horizonte and acts as a reference center for hospitalizations of children and adolescents with infectious diseases. Its patients are referred from the macro-regions of Minas Gerais and from the municipalities of the micro-region and the metropolitan region of Belo Horizonte.^21^ ^,^ ^22^ The coverage of the tetra viral vaccine in the state of Minas Gerais from 2014 to 2016 was 77.8%.^23^

## METHODS

The descriptive and retrospective study was carried out at HIJPII, and the survey of cases was based on the record of hospitalizations for chickenpox that occurred at the hospital, from January 2011 to June 2016, using the International Statistical Classification of Diseases and Health-Related Problems (ICD-10 B01, chickenpox). Convenience sampling was used with all patients who met the inclusion criteria: clinical diagnosis of chickenpox recorded by a pediatrician in the medical record. The exclusion criteria were: the medical records were unavailable or incomplete, the initial suspicion of chickenpox was discarded, and chickenpox was developed during hospitalization (there was no relationship between the disease and the reason for hospitalization). All procedures were approved by the ethics committee of the institution where the authors work (Certificate of Presentation for Ethical Appreciation - *Certificado de Apresentação para Apreciação Ética* CAAE - 53993715.2.0000.5134).

Hospitalizations were classified into pre-vaccine and post-vaccine, considering the date of adding the quadruple viral vaccine to the National Vaccination Calendar: September 2013. The total period analyzed was 66 months: 33 months (January 2011 to September 2013) pre-vaccination and 33 months (October 2013 to June 2016) post-vaccination.

The variables studied were: age and sex of the patients, number of days of hospitalization, causes of hospitalization, need for admission to an intensive care unit (ICU), number of days in the ICU and clinical outcome. The causes of hospitalization were classified into: primary causes, secondary causes, disease in patients at risk and other reasons. The primary causes were defined as disorders caused by the VZV itself and divided into CNS disorders (cerebellitis, VZV encephalitis), febrile seizures and other causes (thrombocytopenia, stomatitis, pneumonitis). Secondary causes were determined as diseases facilitated by the VZV infection and divided into infections of the skin and subcutaneous tissue, respiratory disorders (pneumonia, asthma) and other causes (sepsis, glomerulonephritis, conjunctivitis, arthritis and others). The differential diagnosis between respiratory infections of primary and secondary origin was made based on the patient’s clinical, laboratory and radiological data during hospitalization, according to the assessment of the infectious disease physician.

Hospitalizations were classified as caused by illness in at-risk patients when motivated by infection or by complication in a patient with a condition that decreased their immunity. They were subdivided into malnutrition or chronic diseases (renal, pulmonary, cardiac) and infection from the human immunodeficiency virus (HIV) or immunosuppression (use of corticosteroids in immunosuppressive doses or other immunosuppressors). Hospitalizations for other reasons were those that did not fall into the established categories. Among them include poor general condition, refusal of food, social hospitalization, and others.

Data were collected via medical records, according to a standardized form, and used to create a database in version 22 of the *Statistical Package for the Social Sciences* (SPSS) software (IBM, Armonk, NY, United States ). An analysis of the studied populations was made, comparing data from the pre- and post-vaccination periods. Qualitative variables were displayed as absolute and relative frequencies. Quantitative variables were subjected to the Shapiro-Wilk normality test and presented as mean ± standard deviation when the distribution was normal; and median, minimum and maximum, otherwise. In order to compare qualitative variables between the periods, the proportions and χ^2^ independence tests were adopted. To compare the quantitative variables, the Wilcoxon-Mann-Whitney test was used for independent samples. The relative variation in the number of cases between the two periods was calculated as the difference between the two periods divided by the number of cases in the pre-vaccination period, assessed as a percentage. The analyzes were made in version 3.2.2 of the R program (R Foundation for Statistical Computing, Vienna, Austria), and a significance level of 5% was adopted.

## RESULTS

In the evaluated period, 868 hospitalizations for chickenpox were recorded. In all, 38 patients were excluded from the study due to a lack of information in the medical record or a change in the diagnosis during treatment. The numbers of hospitalizations registered according to the years studied were: 269 (2011); 167 (2012); 233 (2013); 102 (2014); 56 (2015); and three (January to July 2016). A total of 38 cases fit the exclusion criteria. There was a total of 830 hospitalizations for chickenpox, 543 cases in the pre-vaccination period and 287 in the post-vaccination period, with a reduction of 47.1% in hospitalizations (p <0.001). The decrease in hospitalizations by age group was not statistically significant (Table 1).

**Table 1:** Number of hospitalizations for chickenpox, according to age group and their reduction, Hospital Infantil João Paulo II, Minas Gerais.

Age group	Pre-vaccination period n (%)	Post-vaccination period n (%)	Reduction	p-value*
< 1 year	89 (16.4)	45 (15.7)	49.4%	0.869
1 to 5 years	350 (64.5)	185 (64.5)	47.1%	1.000
5 to 10 years	83 (15.3)	49 (17.1)	41.0%	0.569
>10 years	21 (3.9)	8 (2.8)	61.9%	0.544

*p=test of equality of the proportions.

In the total period evaluated, there were more hospitalizations of male individuals (56.1%). The average age of the hospitalized patients was 3.3±2.6 years. Regarding age group, hospitalizations predominated in the age group of one to five year olds(64.5%), followed by children under one year old (16.1%), then, five to ten years old (15.9 %), and last, over ten years old (3.5%).

In the pre-vaccination period, more than half of the hospitalizations involved male individuals (54.7%). The average age of the hospitalized patients during this time was 3.3 ± 2.6 years. In the post-vaccination period, most hospitalizations also occurred with male individuals (58.9%), and was 4.2% higher than in the pre-vaccination period. The average age of the hospitalized patients during this time was 3.4±2.6 years (p=0.267) In both periods, hospitalizations predominated in the age group of children one to five years old (64.5%). There was a decrease in the absolute number of hospitalizations in all age groups in the post-vaccination period, but the percentage of hospitalizations for each age group remained similar (p> 0.05).

Most hospitalizations, in the total period evaluated, were motivated by secondary causes (82.1%). Of these, 91.3% were skin infections. The predominance remained similar in the pre-vaccination and post-vaccination periods. In absolute values, there was a decrease in hospitalizations for primary causes, secondary causes, disease in patients at risk and for other reasons (Tables 2 and 3).

**Table 2:** Causes of hospitalization for patients hospitalized with chickenpox, in the periods before and after the introduction of the chickenpox vaccine, Hospital Infantil João Paulo II, Minas Gerais.

		Pre-vaccination period n (%)	Post-vaccination period n (%)	p-value*
Primary causes	Yes	46 (8.5)	37 (12.9)	0.058
	No	497 (91.5)	250 (87.1)	
Secondary causes	Yes	451 (83.1)	231 (80.5)	0.410
	No	92 (16.9)	56 (19.5)	
Illness in an at-risk patient	Yes	62 (11.4)	35 (12.2)	0.827
	No	481 (88.6)	252 (87.8)	
Other reasons	Yes	20 (3.7)	19 (6.6)	0.084
	No	523 (96.3)	268 (93.4)**	

*p= test χ^2^ of independence; ** percentages calculated on valid cases.

**Table 3:** Causes of hospitalization for patients hospitalized with chickenpox, in the periods before and after the introduction of the chickenpox vaccine, Hospital Infantil João Paulo II, Minas Gerais.

		Pre-vaccination period n (%)	Post-vaccination period n (%)*	p-value**
Primary causes				
CNS disorders	Yes	16 (34.8)	12 (32.4)	1.000
	No	30 (65.2)	25 (67.6)	
Febrile seizure	Yes	16 (34.8)	11 (29.7)	0.801
	No	30 (65.2)	26 (70.3)	
Other causes	Yes	15 (32.6)	14 (37.8)	0.791
	No	31 (67.4)	23 (62.2)	
Secondary causes				
Skin infections	Yes	410 (90.9)	213 (92.2)	0.669
	No	41 (9.1)	18 (7.8)	
Respiratory problems	Yes	49 (10.9)	17 (7.4)	0.184
	No	402 (89.1)	214 (92.6)	
Other causes	Yes	20 (4.4)	14 (6.1)	0.461
	No	431 (95.6)	217 (93.9)	
Risk group				
Chronic diseases and malnutrition	Yes	20 (32.3)	13 (37.1)	0.791
	No	42 (67.7)	22 (62.9)	
Immunosuppression or HIV	Yes	48 (77.4)	27 (77.1)	1.000
	No	14 (22.6)	8 (22.9)	

*Percentages calculated on valid cases; **p=test χ^2^ of independence; CNS: central nervous system; HIV: human immunodeficiency virus.

In the total period evaluated, the median length of stay was 3.9 days (minimum= 0; maximum=97.7); 68 children (8.2%) required ICU admission, with the median time each spent in intensive care being five days (minimum=1; maximum=26). The majority (96.6%) were cured and discharged.

In the total period evaluated, the median length of stay was 4.9 days (minimum=0; maximum=96.6); 44 children (8.1%) required ICU admission, with the median time each spent in intensive care being five days (minimum=1; maximum=26). In the post-vaccination period, the median length of stay was 3.9 days (minimum=0; maximum=34.5; p=0.073), a reduction of approximately one day compared to pre-vaccination; 24 children (8.4%) required admission to the ICU (p = 1.00) - a 45.5% decrease in the absolute number, compared to pre-vaccination. The median length of stay in the ICU was 4.5 days (minimum=2; maximum=15; p=0.096).

In both periods, most children were cured and discharged from the hospital, with a small percentage increase in post-vaccination (1.5%). There was also a reduction in the absolute number of deaths in the period (Table 4).

**Table 4:** Outcomes of hospitalizations for chickenpox, Hospital Infantil João Paulo II, Minas Gerais.

Outcome	Pre-vaccination period n (%)	Post-vaccination period n (%)	Reduction	p-value
Death	6 (1.1)	2 (0.7)	66.7%	0.846
Other (side effect, transfer, other)	15 (2.8)	5 (1.7)	66.7%	
Cured and discharged	522 (96.1)	280 (97.6)	46.4%	

*p=test of equality of the proportions.

## DISCUSSION

The study demonstrated that in the post-vaccination period, there was a substantial drop in hospitalizations for chickenpox, which is in accordance with the national and international medical literature.^9^ ^,^ ^10^ ^,^ ^11^ ^,^ ^12^ ^,^ ^13^ ^,^ ^14^ ^,^ ^15^ ^,^ ^16^ ^,^ ^17^

In Brazil, few studies have evaluated the effects of the varicella vaccine.^17^ ^,^ ^24^ The only one performed nationwide used data from 2003 to 2016, collected through the Department of Informatics of the Public Health System (*Departamento de Informática do Sistema Único de Saúde* - DATASUS). It was found that, after the vaccine was introduced, there was a 47.6% reduction in the number of hospitalizations caused by VZV in the age group of one to four years old, but the study did not differentiate between hospitalizations caused by chickenpox and those caused by the herpes zoster.^17^

Regarding the international literature, most studies relating vaccination and hospitalizations come from the United States, a pioneer in the implementation of universal vaccination with one dose (1995).^9^ Between 1996 and 1997, there was no drop in hospitalizations. ^9^ ^,^ ^25^ Between 1993 and 2001, there was a decrease of 74 (general) and 81.8% (zero to four years of age), considering a vaccination coverage of 76.3%.^9^ ^,^ ^10^ Between 1994 and 2002, there was an 88% decrease, with a predominant decline in children under one year of age.^11^ A two-dose vaccination was introduced in 2006 for children aged four and five. There was a reduction in hospitalizations of 75.6 (1994-2006) and 88.3% (1994-2009).^8^ ^,^ ^12^

The second country to adopt vaccination with one dose was Uruguay (1999). There was a drop in hospitalizations (1999-2005) of 81 (general) and 94% (one to four years of age), considering coverage of 88 to 96%.^9^ ^,^ ^13^ In Australia, which introduced the vaccine at 18 months of age (2005), hospitalizations in the periods from 1999 to 2001 and from 2007 to 2010 fell by 73.2% (p <0.001).^9^ ^,^ ^14^

A literature review described the impact of the vaccine on hospitalization rates associated with chickenpox in countries that adopted universal vaccination between 1995 and May 2015. The study showed that the drop in hospitalizations was repeated, in various ways, in countries like Canada, Spain and Germany.^9^ The variable percentage in the drop in hospitalizations can be explained by the age differences of the populations evaluated, by the time evaluated after the vaccination was introduced, by the regional criteria for hospitalization, by the vaccination coverage, and by the local vaccination strategy.^9^ Thus, it is possible to understand why the data in the literature varies. Thus, it is expected that the drop in hospitalizations in Brazil will be greater in future analyzes, as occurred in the pioneer countries that implemented the vaccine.

The percentage of hospitalizations by age group in the HIJPII was stable. In absolute numbers, there was a decrease in hospitalizations in all age groups. At first, it was expected that the fall would occur exclusively among patients aged 1 to 5 years old, a range that includes children who turned 15 months old in the period evaluated by the study, and who were the target of viral quadruple vaccinations. The decrease in hospitalizations in the age groups not covered by the vaccination is in line with international studies. They demonstrate that vaccination, by reducing the number of infected people, indirectly protects populations that are not eligible for vaccination.^15^ ^,^ ^16^ American studies have verified the incidence of chickenpox in infants outside the vaccine age group (1995-2008) and have shown an 89.7% drop in the incidence of the disease, a number inversely proportional to vaccination coverage, which increased in that period.^15^

The dominant cause of hospitalization in both periods was skin infection (90.9% of cases in the pre-vaccination and 92.2% in the post-vaccination periods). The first American studies also found this cause to be the main cause, affecting 37% of hospitalized children, in both periods.^25^ The predominance was also verified in a study carried out in France (2003-2005), which found skin and soft tissue infections as the cause of 36.5% of pediatric chickenpox admissions in the country.^26^ A study carried out at a referral hospital in Recife found a higher prevalence than in international studies, with 77.3% of the cases that involved complications. This percentage is more consistent with the findings in this study.^27^

The other causes of hospitalization also underwent few percentage changes in the post-vaccination period. The same occurred in the first American studies that evaluated hospitalizations in the post-vaccination period.^25^ In spite of this, all causes of hospitalization suffered a numerical reduction, which was expected considering the global decrease in the number of cases.

The decline in hospitalizations in the risk group is an important point to be discussed. A portion of immunosuppressed patients or those with HIV, such as those that are HIV positive and susceptible to chickenpox and are asymptomatic or oligosymptomatic (categories A1 and N1), were already covered by vaccination in special immunobiological centers before the start of universal vaccination. In addition, individuals susceptible to chickenpox who had home contact with immunocompromised patients were also benefited.^28^ Thus, the drop in hospitalizations in the risk group seems to be related to the increase in the general vaccination coverage of the population, with a consequent decrease in exposure to circulating VZV in the environment (herd immunity).^15^ ^,^ ^16^

Regarding the evolution, it is clear that the length of stay, the need for intensive care and the length of stay in intensive care have undergone little change. These data can be compared, since the service criteria for hospitalization, hospitalization in intensive care, discharge from intensive care and hospital discharge have not changed. As for the outcomes, the vast majority of cases evolved with a cure in both periods. Other outcomes also remained stable. Still, there was a decrease in deaths. Such findings were expected, as the evaluation performed evaluated the pediatric population in general, and not just the vaccinated pediatric population. In vaccinated patients, the literature reports that, if the vaccine fails, they tend to develop milder conditions.^29^ ^,^ ^30^ A recent meta-analysis on the effectiveness of the varicella vaccine showed 81% protection against all forms of the disease and 98% against moderate and severe forms.^29^

The main limitation of this study was the lack of information about the patients’ vaccination status. As such, a description of the populations was made without the results being individualized for vaccinated populations or not. Another limitation was the collection of data in a single service. However, because the HIJPII is the reference hospital for infectious diseases in Minas Gerais, it is believed that the number of hospitalizations in the hospital represents, with good reliability, the population of children hospitalized for chickenpox in the state.

In agreement with the literature, the present study verified the favorable impact of the introduction of the tetra viral vaccine in hospitalizations for varicella. There was a global decrease in the number of hospitalizations, with a numerical reduction of hospitalizations by age group and because of hospitalization. These findings are of great relevance, as they are Brazilian data demonstrating that the measure adopted in the PNI has directly benefited immunized children, and, indirectly, populations not covered by vaccination. Also, with the introduction of the second dose of the chickenpox vaccine in 2018, it is expected that future studies will identify an even greater reduction in hospitalizations caused by the disease.

## References

[1] Brazil - Ministério da Saúde. Secretaria de Vigilância em Saúde.
Coordenação-Geral de Desenvolvimento da Epidemiologia em
Serviços (2019). Guia de vigilância em saúde: volume único.

[2] Whitley RJ, Kasper DL (2017). Infecções pelo vírus varicela zóster. Medicina interna de Harrison.

[3] Berezin EN, Feldman C, Focaccia R, Veronesi R (2015). Varicela-zoster. Tratado de infectologia.

[4] [No authors listed] (2014). Varicella and herpes Zoster vaccines. WHO position paper, June
2014. Wkly Epidemiol Rec.

[5] Brazil - Ministério da Saúde Portal da Saúde. Situação epidemiológica.

[6] European Centre for Disease Prevention and Control (2015). Varicella vaccination in the European Union.

[7] Seward JF, Marin M (2014). Varicella Disease burden and varicella vaccines. Proceedings of the WHO SAGE Meeting.

[8] Lozano R, Naghavi M, Foreman K, Lim S, Shibuya K, Aboyans V (2012). Global and regional mortality from 235 causes of death for 20 age
groups in 1990 and 2010: a systematic analysis for the Global Burden of
Disease Study 2010. Lancet.

[9] Hirose M, Gilio AE, Ferronato AE, Ragazzi SL (2016). The impact of varicella vaccination on varicella-related
hospitalization rates: global data review. Rev Paul Pediatr.

[10] Davis MM, Patel MS, Gebremariam A (2004). Decline in varicella-related hospitalizations and expenditures
for children and adults after introduction of varicella vaccine in the
United States. Pediatrics.

[11] Zhou F, Harpaz R, Jumaan AO, Winston CA, Shefer A (2005). Impact of varicella vaccination on health care
utilization. JAMA.

[12] Baxter R, Tran TN, Ray P, Lewis E, Fireman B, Black S (2014). Impact of vaccination on the epidemiology of varicella:
1995-2009. Pediatrics.

[13] Quian J, Ruttimann R, Romero C, Dall’Orso P, Cerisola A, Breuer T (2008). Impact of universal varicella vaccination on 1-year-olds in
Uruguay: 1997-2005. Arch Dis Child.

[14] Marshall HS, McIntyre P, Richmond P, Buttery JP, Royle JA, Gold MS (2013). Changes in patterns of hospitalized children with varicella and
of associated varicella genotypes after introduction of varicella vaccine in
Australia. Pediatr Infect Dis J.

[15] Chaves SS, Lopez AS, Watson TL, Civen R, Watson B, Mascola L (2011). Varicella in infants after implementation of the US varicella
vaccination program. Pediatrics.

[16] Singleton RJ, Holman RC, Person MK, Steiner CA, Redd JT, Hennessy TW (2014). Impact of varicella vaccination on varicella-related
hospitalizations among American Indian/Alaska native people. Pediatr Infect Dis J.

[17] Scotta MC, Paternina-de la Ossa R, Lumertz MS, Jones MH, Mattiello R, Pinto LA (2018). Early impact of universal varicella vaccination on childhood
varicella and herpes zoster hospitalizations in Brazil. Vaccine.

[18] Brazil - Ministério da Saúde Portal da Saúde. Calendário Nacional de Vacinação 2019.

[19] Minas Gerais. Governo do Estado de Minas Gerais. Secretaria de
Estado da Saúde. Subsecretaria de Vigilância e Proteção à Saúde (2013). Protocolo de Varicela.

[20] Valentim J, Sartori AM, Soárez PC, Amaku M, Azevedo RS, Novaes HM (2008). Cost-effectiveness analysis of universal childhood vaccination
against varicella in Brazil. Vaccine.

[21] Fundação Hospitalar do Estado de Minas Gerais - FHEMIG Hospital Infantil Joao Paulo II.

[22] Fundação Hospitalar do Estado de Minas Gerais - FHEMIG Atendimento médico a doenças infecto-contagiosas
infanto-juvenis.

[23] Brazil - Ministério da Saúde - DATASUS Imunizações- cobertura- Brasil.

[24] Kupek E, Tritany EF (2009). Impact of vaccination against varicella on the reduction of the
disease incidence in children and adolescents from Florianópolis,
Brazil. J Pediatr (Rio J).

[25] Rhein L, Fleisher GR, Harper MB (2001). Lack of reduction in hospitalizations and emergency department
visits for varicella in the first 2 years post-vaccine
licensure. Pediatr Emerg Care.

[26] Grimprel E, Levy C, de La Rocque F, Cohen R, Soubeyrand B, Caulin E (2007). Paediatric varicella hospitalisations in France: a nationwide
survey. Clin Microbiol Infect.

[27] Anjos KS, Ferreira MM, Arruda MC, Ramos KS, Magalhães AP (2009). Epidemiological characterization of varicella cases in patients
of a university hospital located in Recife. Rev Bras Epidemiol.

[28] Brazil - Ministério da Saúde. Secretaria de Vigilância em Saúde.
Departamento de Vigilância Epidemiológica (2006). Manual dos centros de referência para imunobiológicos especiais.

[29] Marin M, Marti M, Kambhampati A, Jeram SM, Seward JF (2016). Global varicella vaccine effectiveness: a
meta-analysis. Pediatrics.

[30] Andrade AL, da Silva Vieira MA, Minamisava R, Toscano CM, de Lima Souza MB, Fiaccadori F (2018). Single-dose varicella vaccine effectiveness in Brazil: a
case-control study. Vaccine.

