# ERRATUM

**DOI:** 10.1590/1984-0462/2021/39/2019215erratum

**Published:** 2020-10-21

In the manuscript “Number of cases of varicella and hospitalization in a pediatric reference hospital in Brazil after introducing the vaccine”, DOI: http://dx.doi.org/10.1590/1984-0462/2021/39/2019215, published in the Rev. Paul. pediatr. [Internet]. 2020;39:e2019215. Epub June 26, 2020, in page 1.

**Where it reads:**

Henrique Botelho de Abreu e Silva^a^ , Isabella Cristina Tristão Pinto^a^ , José Geraldo Leite Ribeiro^a^ , Leonardo Santos Resende^a^ , Ludymila Keren de Carvalho^a^ , Marcelle Marie Martins Maia^b^ , Roberto Ferreira de Almeida Araújo^a^ , Lilian Martins Oliveira Diniz^c,^*

^a^Faculdade Ciências Médicas de Minas Gerais, Belo Horizonte, MG, Brazil.

^b^School of Medicine, Universidade Federal de Minas Gerais, Belo Horizonte, MG, Brazil.

^c^Hospital Infantil João Paulo II, Belo Horizonte, MG, Brazil.

**It should read:**

Isabella Cristina Tristão Pinto^a^ , Lilian Martins Oliveira Diniz^b,*^ , Ludymila Keren de Carvalho^a^ , Leonardo Santos Resende^a^ , Henrique Botelho de Abreu e Silva^a^ , Roberto Ferreira de Almeida Araújo^a^ , Marcelle Marie Martins Maia^c^ , José Geraldo Leite Ribeiro^a^

^a^Faculdade Ciências Médicas de Minas Gerais, Belo Horizonte, MG, Brazil.

^b^Hospital Infantil João Paulo II, Belo Horizonte, MG, Brazil.

^c^School of Medicine, Universidade Federal de Minas Gerais, Belo Horizonte, MG, Brazil.

